# The Price of Gas

**Published:** 1916-03-11

**Authors:** W. M. Mason

**Affiliations:** Secretary. The British Commercial Gas Association, 47 Victoria Street, Westminster, S.W.


### The Price of Gas.

*To the Editor of* The Hospital.

Sir,—In the issue of the *Institutional Worker* for February 26, I see that it is stated under the heading of " Hospital Fuel and Lighting " that it is doubtful whether the prices now paid for coal, coke, gas, etc., have previously been equalled."

You will, therefore, I am sure, be interested to know that in the district of the largest gas undertaking in the country—the Gas Light and Coke Company, of London—the price now charged for gas, although 6d. per 1,000 cubic feet higher than before the outbreak of war, is still 5d. per 1,000 cubic feet below the level to which it rose at the last period of coal scarcity—namely, 1901—when the price was for a considerable time 3s. 5d. per 1,000 cubic feet.

To a greater or less extent this is true of most of the gas undertakings up and down the country, as the steady growth of the use of gas for fuel in recent years, and the introduction of modern methods of manufacture, distribution, and sale, have combined to bring about a steady reduction in the cost of gas to the public.

Whilst, therefore, it is true that coal is probably considerably dearer than at any previous time within average memory, gas is still at a very reasonable level, and that, no doubt, accounts for the very rapid growth in the use of gas as a fuel by the general public, and specially by hospitals and other institutions, since the outbreak of war.

This has the patriotic advantage of enabling the gas industry to maintain at a fair, though even now none too high a level, its output of raw materials for high explosives, which are a by-product of gas manufacture.

It may be not out of place to remind your readers that the public are safeguarded against any undue rise in the price of gas by the fact that, unlike any other industry, the dividends payable to the shareholders are automatically reduced immediately the price of gas is increased, and *vice versa*.

The consumers of gas and the shareholders in gas companies are therefore in partnership, benefiting mutually by any savings in manufacturing or working costs, or by a favourable condition of the markets, and suffering mutually when conditions are adverse.—Yours faithfully,

W. M. Mason, Secretary.

The British Commercial Gas Association,
47 Victoria Street, Westminster, S.W.
February 29, 1916.

